# Supplementary material for: Transport and inhibition of the sphingosine-1-phosphate exporter SPNS2
Source: Nat Commun. 2025 Jan 16;16:721. doi: 10.1038/s41467-025-55942-7 (PMC11739509; doi:10.1038/s41467-025-55942-7)
Supplement: Supplementary file 2 — Reporting Summary [file 41467_2025_55942_MOESM2_ESM.pdf]

nature portfolio

Corresponding author(s): Katharina L. Dürr, Vera Puetter, Alexander Ehrmann, Syma Khalid, Alvaro Ingles-Prieto, Giulio Superti-Furga, David B. Sauer

Last updated by author(s): 11/27/2024

Reporting Summary

Nature Portfolio wishes to improve the reproducibility of the work that we publish. This form provides structure for consistency and transparency in reporting. For further information on Nature Portfolio policies, see our Editorial Policies and the Editorial Policy Checklist.

Statistics

For all statistical analyses, confirm that the following items are present in the figure legend, table legend, main text, or Methods section.

n/a	Confirmed	
☐	☒	The exact sample size (*n*) for each experimental group/condition, given as a discrete number and unit of measurement
☐	☒	A statement on whether measurements were taken from distinct samples or whether the same sample was measured repeatedly
☒	☐	The statistical test(s) used AND whether they are one- or two-sided<br>*Only common tests should be described solely by name; describe more complex techniques in the Methods section.*
☒	☐	A description of all covariates tested
☒	☐	A description of any assumptions or corrections, such as tests of normality and adjustment for multiple comparisons
☒	☐	A full description of the statistical parameters including central tendency (e.g. means) or other basic estimates (e.g. regression coefficient) AND variation (e.g. standard deviation) or associated estimates of uncertainty (e.g. confidence intervals)
☒	☐	For null hypothesis testing, the test statistic (e.g. *F*, *t*, *r*) with confidence intervals, effect sizes, degrees of freedom and *P* value noted<br>*Give P values as exact values whenever suitable.*
☒	☐	For Bayesian analysis, information on the choice of priors and Markov chain Monte Carlo settings
☒	☐	For hierarchical and complex designs, identification of the appropriate level for tests and full reporting of outcomes
☒	☐	Estimates of effect sizes (e.g. Cohen's *d*, Pearson's *r*), indicating how they were calculated

Our web collection on statistics for biologists contains articles on many of the points above.

Software and code

Policy information about availability of computer code

Data collection	Cryo-EM data were collected with EPU (version 2.13; Thermo Fisher Scientific).
Data analysis	Cryo-EM data were processed and analyzed with cryoSPARC (version 3.3.1; Structura Biotechnology). Additional software from the Collaborative Computational Projects No. 4 (CCP4; v7.0) and Electron cryo-Microscopy (CCPEM; v1.6.0) software suites were also used. Structural models were built using Coot (v0.8.9.5) with model refinement performed by PHENIX (v1.20).<br><br>Molecular dynamics simulations were performed using GROMACS 2020.3 simulation suite (Royal Institute of Technology and Uppsala University, Sweden).<br><br>Molecular visualisation, analysis and figure generation were performed using PyMOL 2.2.0 (Schrodiner), Visual Molecular Dynamics 1.9.4 (University of Illinois Urbana-Champagne), and ChimeraX 1.16 (UCSF Resource for Biocomputing, Visualization, and Informatics).

For manuscripts utilizing custom algorithms or software that are central to the research but not yet described in published literature, software must be made available to editors and reviewers. We strongly encourage code deposition in a community repository (e.g. GitHub). See the Nature Portfolio guidelines for submitting code & software for further information.

Data

Policy information about availability of data

All manuscripts must include a data availability statement. This statement should provide the following information, where applicable:

- Accession codes, unique identifiers, or web links for publicly available datasets
- A description of any restrictions on data availability
- For clinical datasets or third party data, please ensure that the statement adheres to our policy

The cryo-EM maps have been deposited in the Electron Microscopy Data Bank (EMDB) under accession codes EMD-18668 (DDM solubilized) and EMD-18667 (LMNG solubilized). The atomic models have been deposited in the Protein Data Bank under accession codes 8QV6 (DDM solubilized) and 8QV5 (LMNG solubilized).

Research involving human participants, their data, or biological material

Policy information about studies with human participants or human data. See also policy information about sex, gender (identity/presentation), and sexual orientation and race, ethnicity and racism.

Reporting on sex and gender	NA
Reporting on race, ethnicity, or other socially relevant groupings	NA
Population characteristics	NA
Recruitment	NA
Ethics oversight	NA

Note that full information on the approval of the study protocol must also be provided in the manuscript.

Field-specific reporting

Please select the one below that is the best fit for your research. If you are not sure, read the appropriate sections before making your selection.

☒ Life sciences ☐ Behavioural & social sciences ☐ Ecological, evolutionary & environmental sciences

For a reference copy of the document with all sections, see nature.com/documents/nr-reporting-summary-flat.pdf

Life sciences study design

All studies must disclose on these points even when the disclosure is negative.

Sample size	No statistical methods were used to predetermine sample sizes as this manuscript does not contain data for which statistical analysis was required.
Data exclusions	No data was excluded during this study.
Replication	Cryo-EM data collection was only carried out on a single sample in each case.<br><br>All in vivo experiments used either 4, 8, or 24 biological replicates.<br><br>In vitro binding assays were carried out with 3 biological replicates.<br><br>Molecular dynamics simulations used 5 independent runs for each substrate condition.<br><br>All replicates were successful and no data was excluded from this study.
Randomization	This is not relevant for our study. Each replicate is an independent experiment using defined reagents.
Blinding	Blinding was not used. It is not relevant as outcomes of the experiments are not affected by knowledge of the sample.

Reporting for specific materials, systems and methods

We require information from authors about some types of materials, experimental systems and methods used in many studies. Here, indicate whether each material, system or method listed is relevant to your study. If you are not sure if a list item applies to your research, read the appropriate section before selecting a response.

Materials & experimental systems

n/a	Involved in the study	
☐	☒	Antibodies
☐	☒	Eukaryotic cell lines
☒	☐	Palaeontology and archaeology
☐	☒	Animals and other organisms
☒	☐	Clinical data
☒	☐	Dual use research of concern
☒	☐	Plants

Methods

n/a	Involved in the study	
☒	☐	ChIP-seq
☒	☐	Flow cytometry
☒	☐	MRI-based neuroimaging

Antibodies

Antibodies used: NbD12 and NbF09

Validation: Nanobodies were validated by immunofluoresence, biolayer interferometry, Co-IP, and structural studies, as described in the manuscript.

Conditions of use for all nanobodies is described in the methods.

Eukaryotic cell lines

Policy information about cell lines and Sex and Gender in Research

Cell line source(s): Spodoptera frugiperda (Sf9) cells (Thermo-Fisher Scientific, Cat. No. 11496015). Expi293F GnTI- cells (Cat# A39240; Thermo Fisher Scientific). Jump In T-REx human embryonic kidney 293 (HEK293-JI) cells (Cat# A15008; Thermo Fisher Scientific).

Authentication: Cell lines used (SF9/Expi293F/HEK293-JI) are standard laboratory model overexpression strains purchased from Thermo Fisher. These cell lines undergo quality control before dispatch. Cells were passaged a limited number of times before a new batch from the manufacturer was employed. Cells were monitored by regular visual inspection.

Mycoplasma contamination: N/A

Commonly misidentified lines (See ICLAC register): N/A

Animals and other research organisms

Policy information about studies involving animals; ARRIVE guidelines recommended for reporting animal research, and Sex and Gender in Research

Laboratory animals: Adult Alpaca (Vicugna pacos)

Wild animals: N/A

Reporting on sex: Sex is not relevant to results as the alpaca were used only for nanobody generation.

Field-collected samples: N/A

Ethics oversight: The immunizations of alpaca were conducted strictly according to the guidelines of the Swiss Animals Protection Law and were approved by the Cantonal Veterinary Office of Zurich, Switzerland (Licenses No. ZH 198/17 and ZH028/2021).

Note that full information on the approval of the study protocol must also be provided in the manuscript.

Plants

Seed stocks	*Report on the source of all seed stocks or other plant material used. If applicable, state the seed stock centre and catalogue number. If plant specimens were collected from the field, describe the collection location, date and sampling procedures.*
Novel plant genotypes	*Describe the methods by which all novel plant genotypes were produced. This includes those generated by transgenic approaches, gene editing, chemical/radiation-based mutagenesis and hybridization. For transgenic lines, describe the transformation method, the number of independent lines analyzed and the generation upon which experiments were performed. For gene-edited lines, describe the editor used, the endogenous sequence targeted for editing, the targeting guide RNA sequence (if applicable) and how the editor was applied.*
Authentication	*Describe any authentication procedures for each seed stock used or novel genotype generated. Describe any experiments used to assess the effect of a mutation and, where applicable, how potential secondary effects (e.g. second site T-DNA insertions, mosiacism, off-target gene editing) were examined.*
