# Supplementary material for: Transport and inhibition of the sphingosine-1-phosphate exporter SPNS2
Source: Nat Commun. 2025 Jan 16;16:721. doi: 10.1038/s41467-025-55942-7 (PMC11739509; doi:10.1038/s41467-025-55942-7)
Supplement: Supplementary file 4 — Source Data [file 41467_2025_55942_MOESM4_ESM.zip › Source Data/source_fig1_27aug2024.pdf]

a

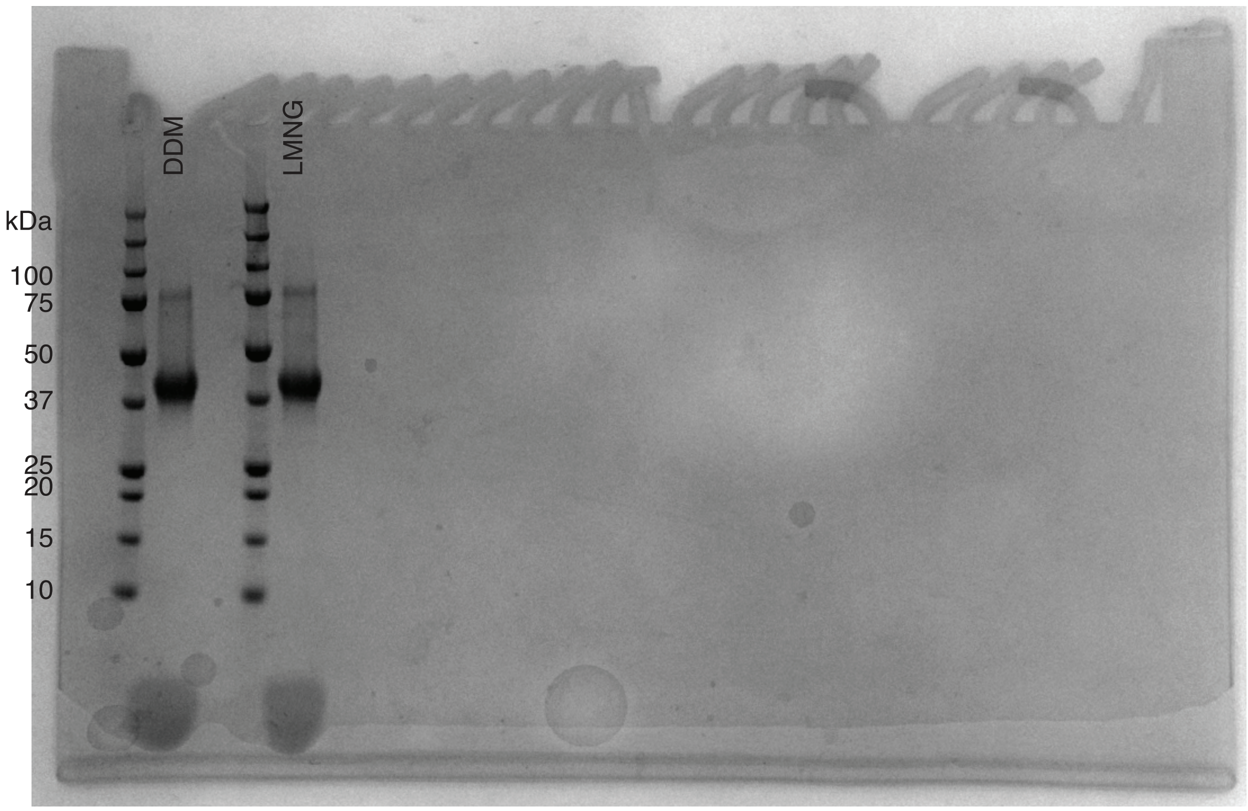

b

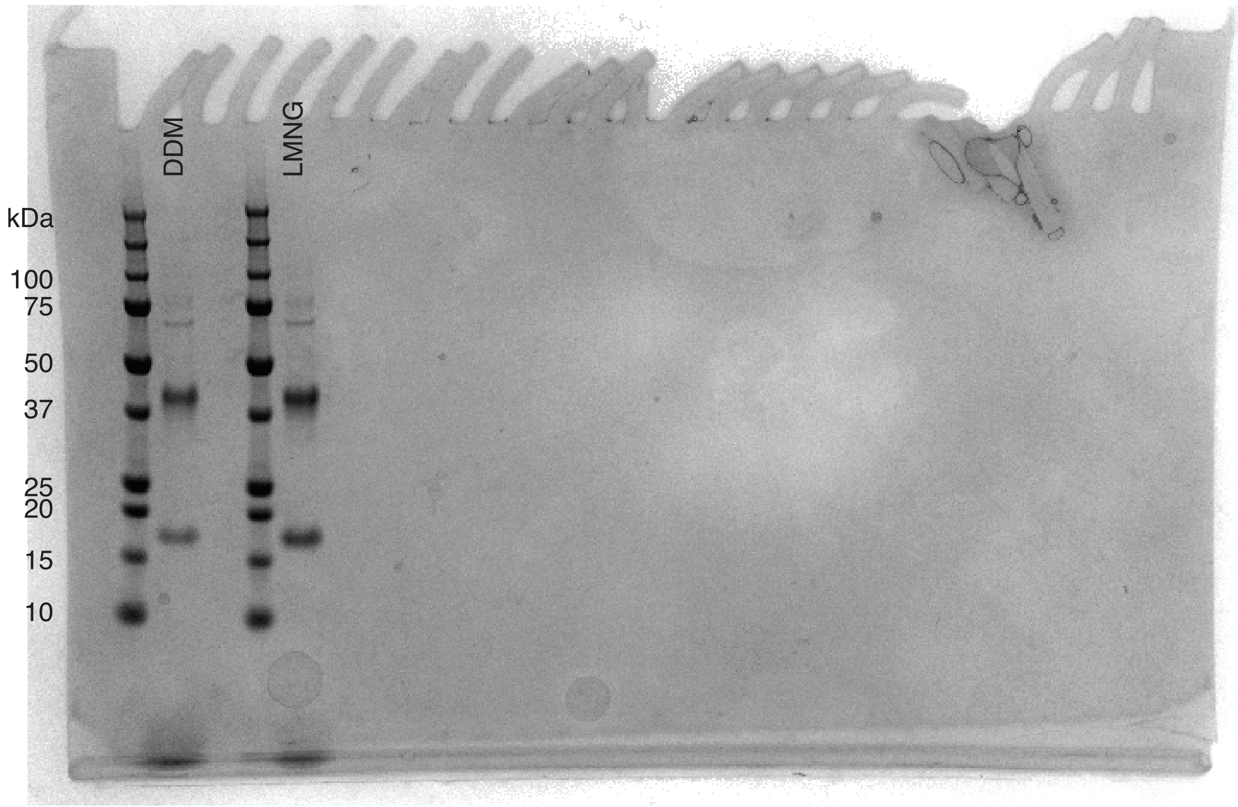

Source Data 1. SPNS2 and SPNS2-NbD12 complex purifications. a Uncropped SDS-PAGE gel with Coomassie stain of SPNS2 purified in (left) DDM and (right) LMNG. **b** Uncropped SDS-PAGE gel with Coomassie stain of SPNS2-NbD12 complex purified in (left) DDM and (right) LMNG.
