# Supplementary material for: Transport and inhibition of the sphingosine-1-phosphate exporter SPNS2
Source: Nat Commun. 2025 Jan 16;16:721. doi: 10.1038/s41467-025-55942-7 (PMC11739509; doi:10.1038/s41467-025-55942-7)
Supplement: Supplementary file 1 — Supplementary Information [file 41467_2025_55942_MOESM1_ESM.pdf]

Supplementary information for

Transport and inhibition of the sphingosine-1-phosphate exporter SPNS2

Huanyu Z. Li [1], Ashley C.W. Pike [1*], Yung-Ning Chang [2*], Dheeraj Prakaash [3*], Zuzana Gelova [4*], Josefina Stanka [5*], Christophe Moreau [1], Hannah C. Scott [1,6], Frank Wunder [5], Gernot Wolf [4], Andreea Scacioc [1], Gavin McKinley [1], Helena Batoulis [5], Shubhashish Mukhopadhyay [1], Andrea Garofoli [4], Adán Pinto-Fernández [1,6], Benedikt M. Kessler [1,6], Nicola A. Burgess-Brown [1], Saša Štefanić [7], Tabea Wiedmer [4], Katharina L. Dürr [1†], Vera Puetter [2†], Alexander Ehrmann [5†], Syma Khalid [3†], Alvaro Ingles-Prieto [4†], Giulio Superti-Furga [4,8†], David B. Sauer [1†]

1 Centre for Medicines Discovery, Nuffield Department of Medicine, University of Oxford, Oxford, UK

2 Nuvisan ICB GmbH, Berlin, Germany

3 Department of Biochemistry, University of Oxford, Oxford, UK

4 CeMM Research Center for Molecular Medicine of the Austrian Academy of Sciences, Vienna, Austria

5 Bayer AG

6 Chinese Academy for Medical Sciences Oxford Institute, Nuffield Department of Medicine, University of Oxford, Oxford, UK

7 Nanobody Service Facility, University of Zurich, AgroVet-Strickhof, Eschikon, Switzerland

8 Center for Physiology and Pharmacology, Medical University of Vienna, Vienna, Austria

[*] Authors contributed equally: A.C.W.P., Y.N.C., D.P., Z.G., J.S.

[†] Correspondence to: K.L.D. (katharina.duerr@omass.com), V.P. (Vera.Puetter@nuvisan.com), A.E. (alexander.ehrmann@bayer.com), S.K. (syma.khalid@bioch.ox.ac.uk), A.I.P. (ingles.prieto@gmail.com), G.S.F. (GSuperti@cemm.oeaw.ac.at), D.B.S. (david.sauer@cmd.ox.ac.uk)

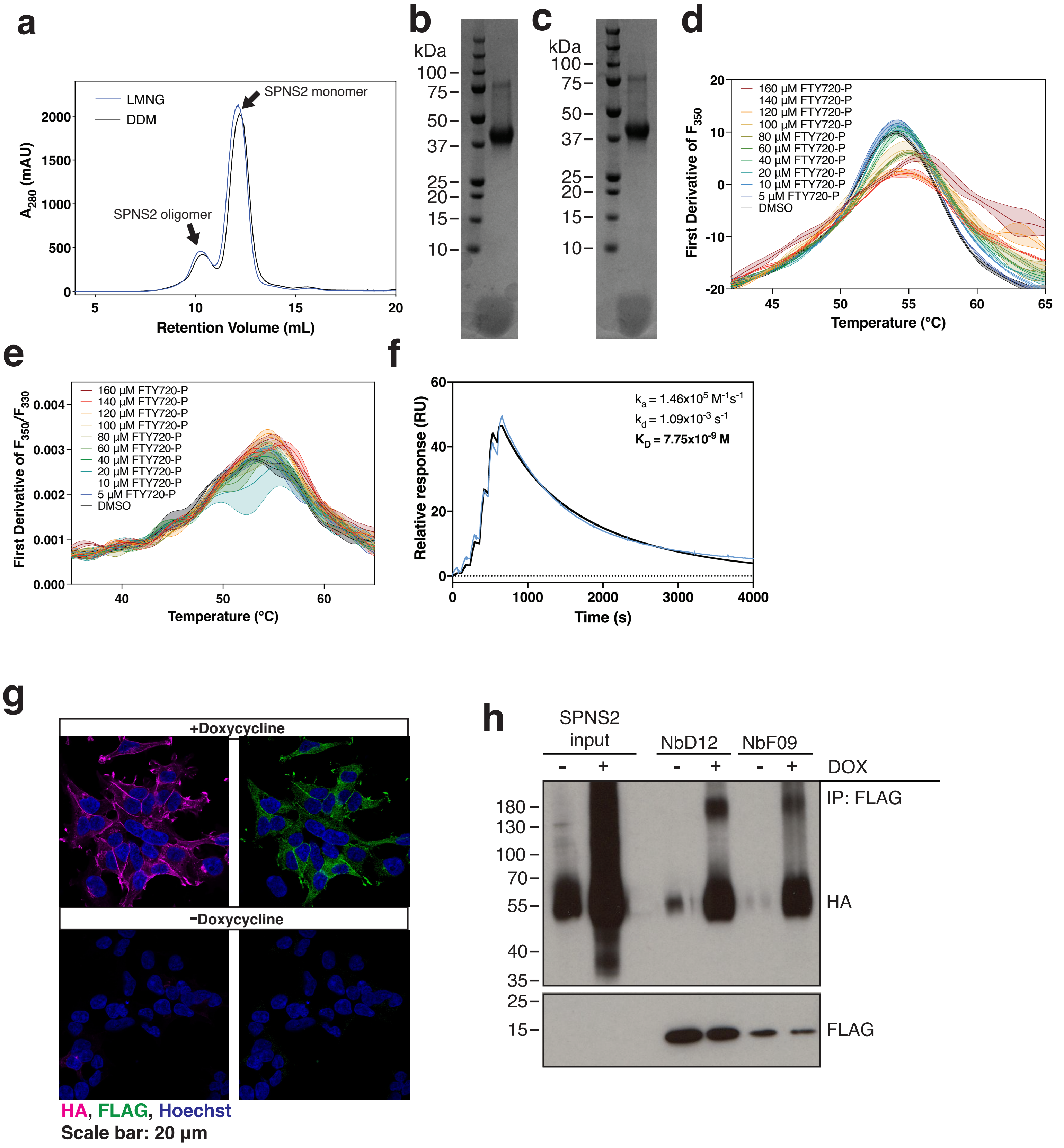

Supplementary Figure 1. Purification of SPNS2 and SPNS2-Nb complexes. a Preparative size-exclusion chromatography of SPNS2 in DDM or LMNG on a Superdex 200 Increase 10/300 GL column. Peaks for momeric and oligomeric SPNS2 are assigned based on retention volume. Representative SDS-PAGE of SPNS2 purification from 10 biological replicates in (**b**) DDM and (**c**) LMNG. **d** Thermostabilization of DDM solubilized SPNS2 by FTY720-P (N = 3). Data are presented as mean ± SEM. **e** Thermostabilization of LMNG solubilized the voltage-gated purine nucleotide uniporter by FTY720-P (N = 3). Data are presented as mean ± SEM. **f** Surface Plasmon Resonance of NbD12 binding to DDM-solubilized SPNS2. **g** Immunofluorescence of HEK293 cells with and without SPNS2 expression induced with doxycycline and stained with NbD12. **h** Immunoprecipatation using FLAG-tagged NbD12 and NbF09 of HEK293 cells expressing HA-tagged SPNS2, and stained with anti-HA and anti-FLAG antibodies.

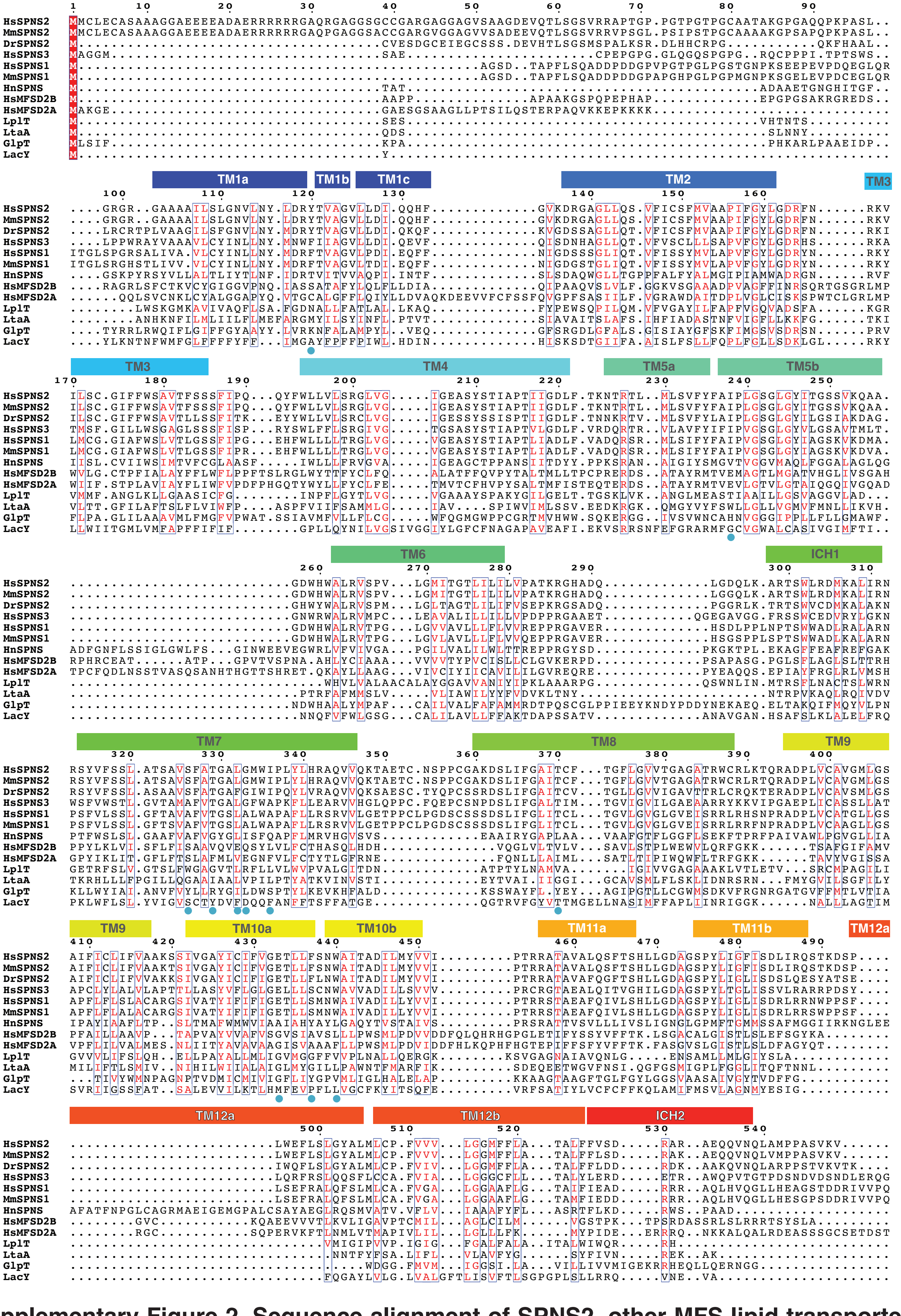

Supplementary Figure 2. Sequence alignment of SPNS2, other MFS lipid transporters, and prototype MFS proteins. Transmembrane helices of HsSPNS2 and amino acids coordinating the DDM are indicated by colored bars above and cyan circles below, respectively.

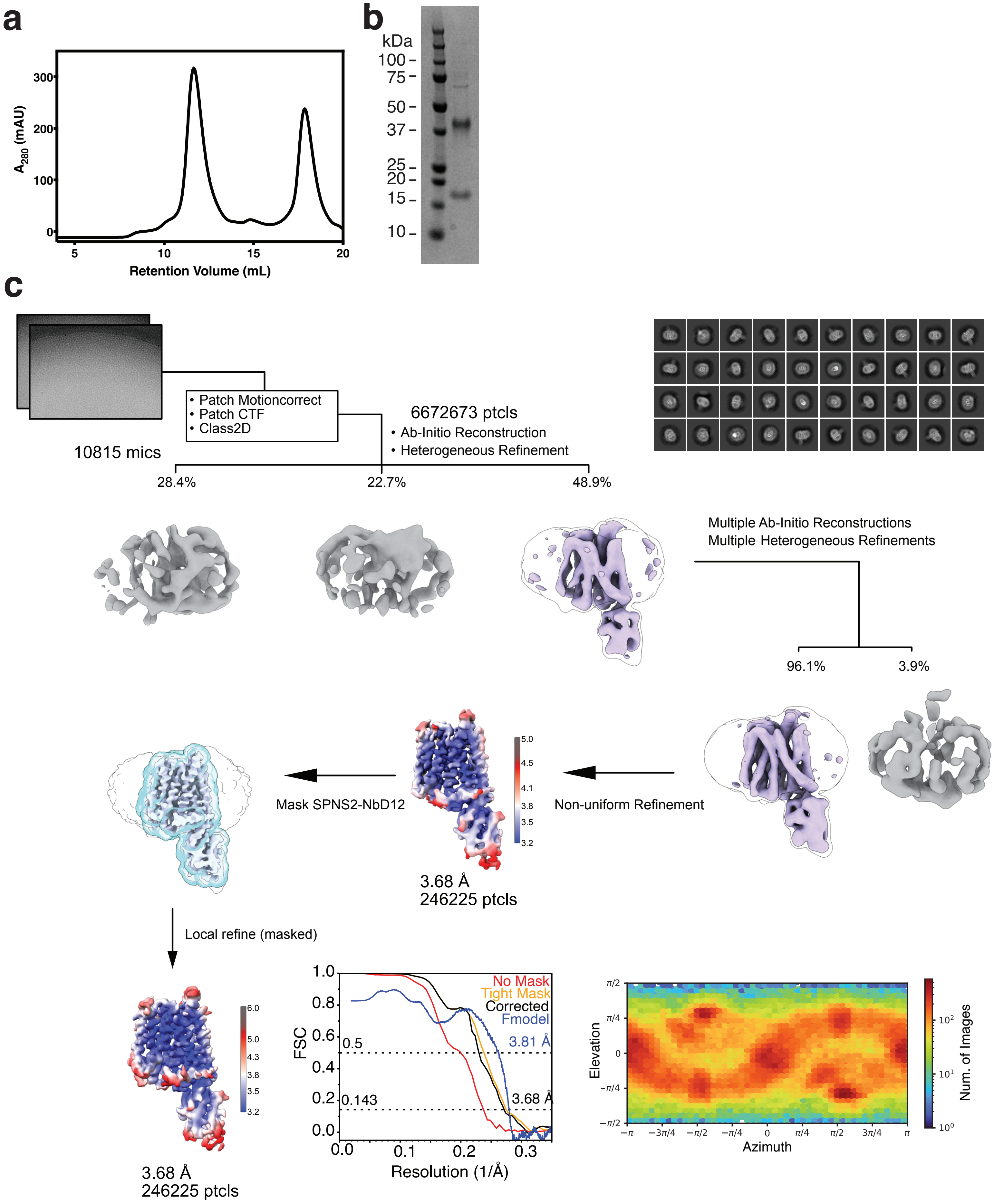

Supplementary Figure 3. Structure determination of SPNS2 in DDM. a Preparative size-exclusion chromatography of SPNS2 in complex with NbD12 in DDM on a Superdex 200 Increase 10/300 GL column. **b** Representative SDS-PAGE of SPNS2-NbD12 complex purification in DDM from 10 biological replicates. **c** Flow chart for determining the structure of the SPNS2-NbD12 complex in DDM.

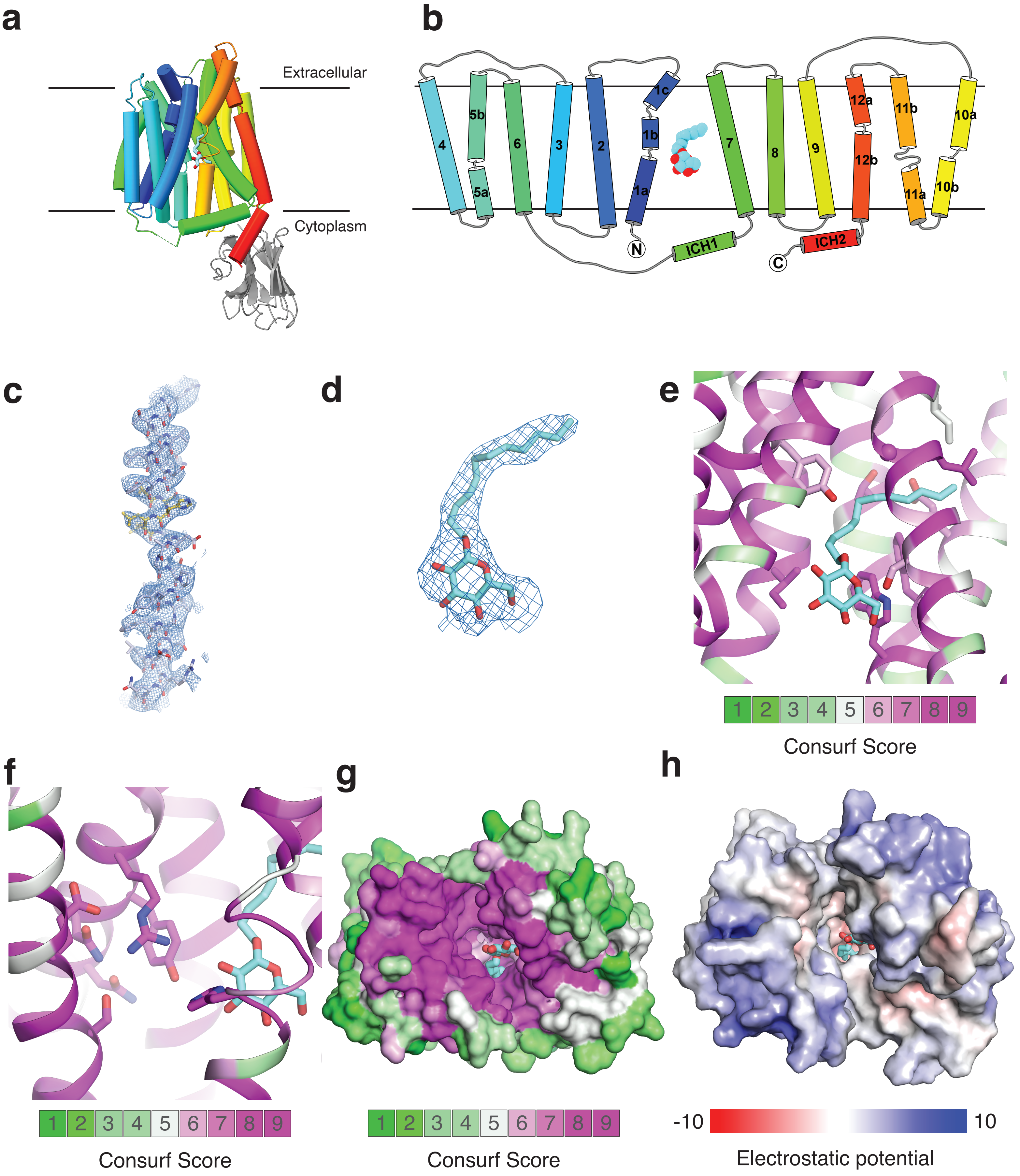

Supplementary Figure 4. Structure and conservation of SPNS2. a Structure of SPNS2 in DDM. SPNS2 and NbD12 are colored in rainbow and grey, respectively. **b** Topology of SPNS2. Structure and Coulombic potential map of the (**c**) pi-helix within TM11 and (**d**) DDM molecule in the binding site. **e** Sequence conservation of the SPNS2 pocket. **f** Sequence conservation of SPNS2 polar patch. **g** Sequence conservation of SPNS2 viewed from the cytoplasm. **h** Electrostatic potential of SPNS2 viewed from the cytoplasm.

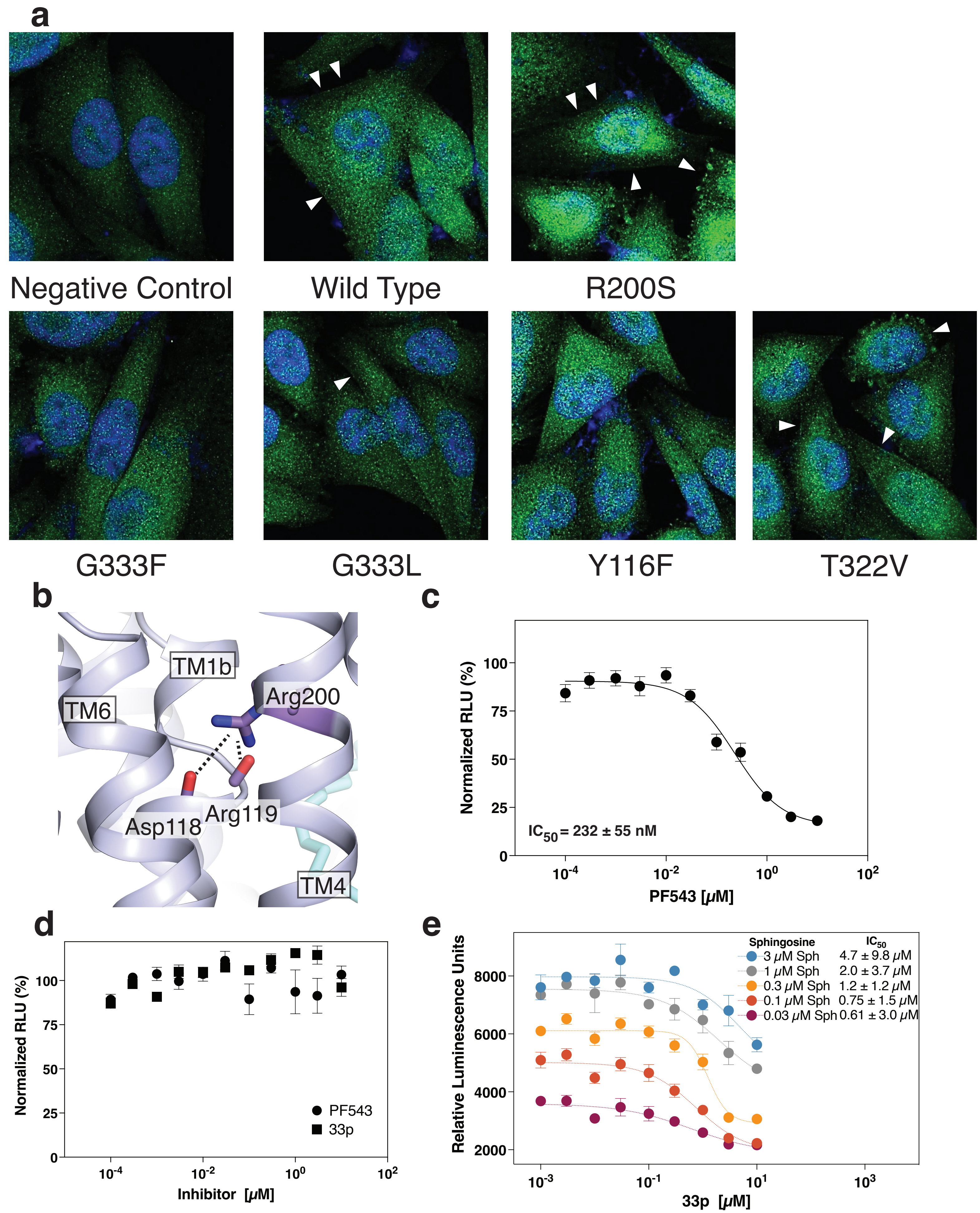

Supplementary Figure 5. Localization and structural effects of SPNS2 mutants. a Anti-FLAG immunofluorescence images of SPNS2 and its mutants, stained with FLAG-tagged NbF09 and DAPI. Arrows highlight plasma membrane staining with anti-SPNS2 NbF09. **b** Local structure and interactions of Arg200 within SPNS2. **c** Inhibition of S1P export by the SphK1 inhibitor PF543 (N = 8). Data are presented as mean ± SEM. **d** S1PR3 signalling in response to PF543 and 33p (N = 8). **e** Inhibition of S1P export by 33p with varying external sphingosine (N = 4).

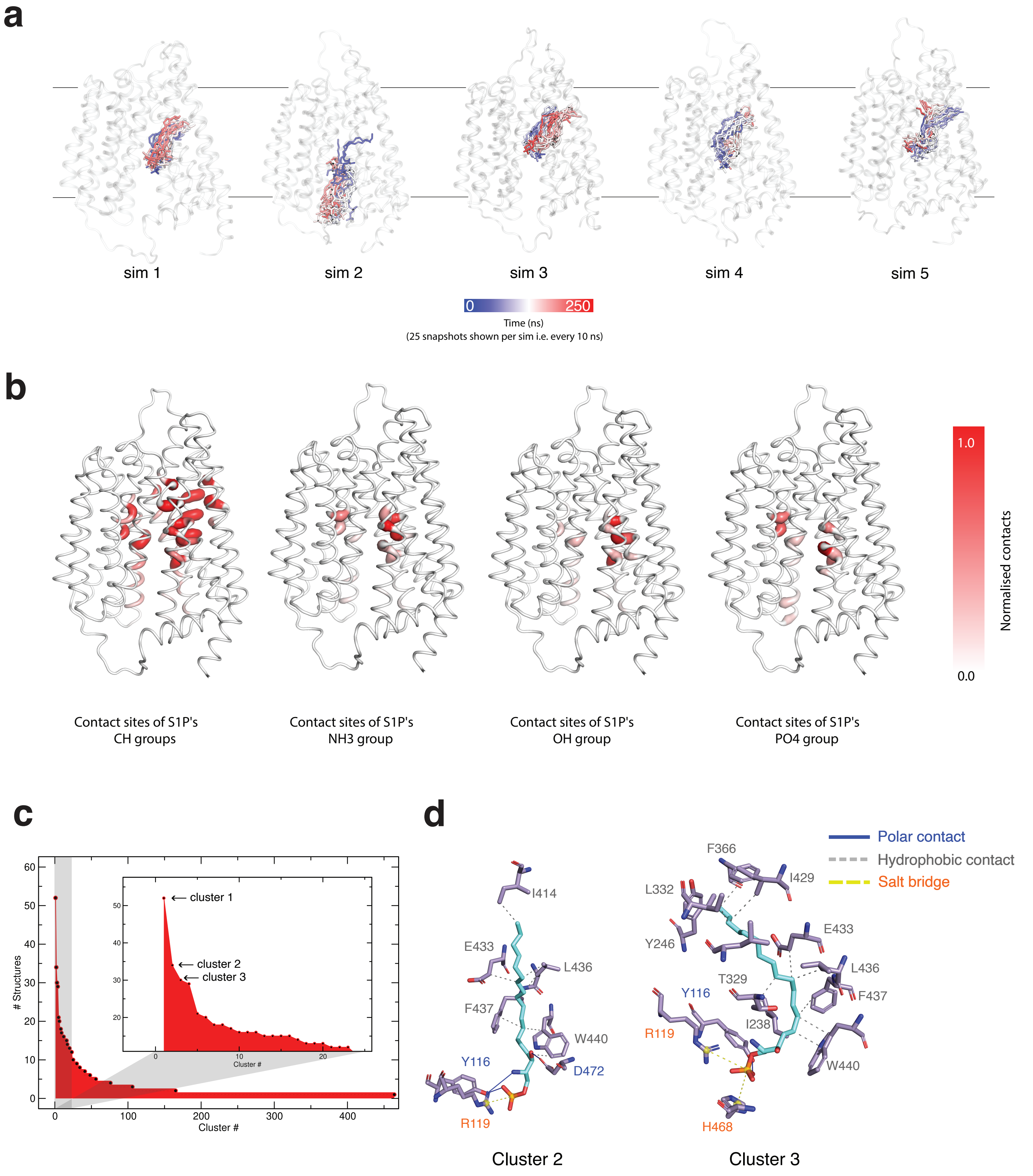

Supplementary Figure 6. Molecular dynamics of SPNS2 bound to S1P. a Time course of S1P location and pose for 5 simulations with 25 snapshots per simulations. **b** Frequency of contacts for residues within 4 Å of SPNS2. **c** Histogram of SPNS2 with S1P structural clusters. SPNS2 snapshots are clustered if within 1 Å RMSD. **d** S1P interactions with SPNS2 in clusters 2 and 3. Carbons of SPNS2 side chains and S1P are shown in purple and cyan, respectively. Interaction types are annotated by PLIP.

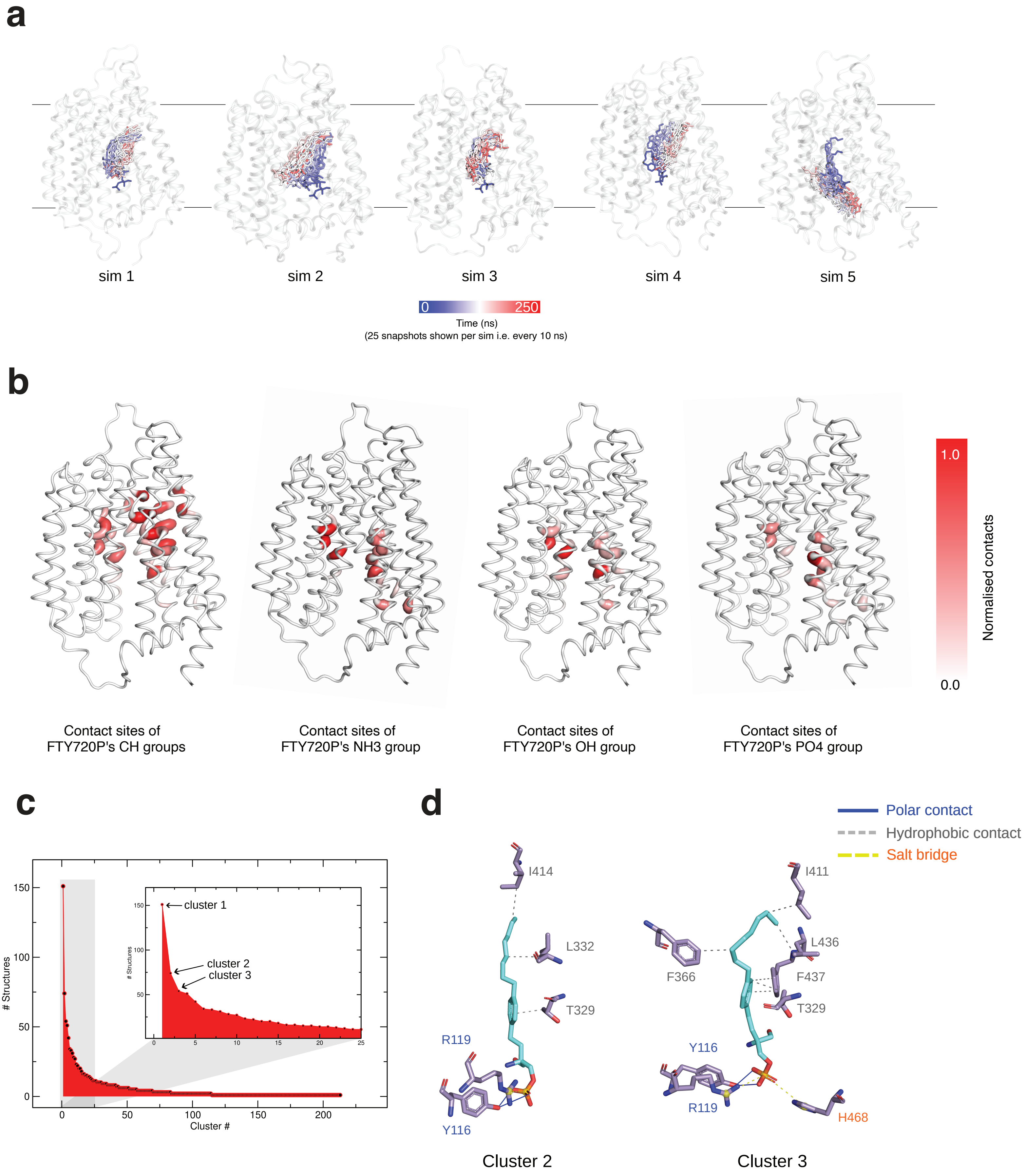

Supplementary Figure 7. Molecular dynamics of SPNS2 bound to FTY720-P. **a** Time course of FTY720-P location and pose for 5 simulations with 25 snapshots per simulations. **b** Frequency of contacts for residues within 4 Å of SPNS2. **c** Histogram of SPNS2 with FTY720-P structural clusters. SPNS2 snapshots are clustered if within 1 Å RMSD. **d** FTY720-P interactions with SPNS2 in clusters 2 and 3. Carbons of SPNS2 side chains and FTY720-P are shown in purple and cyan, respectively. Interaction types are annotated by PLIP.

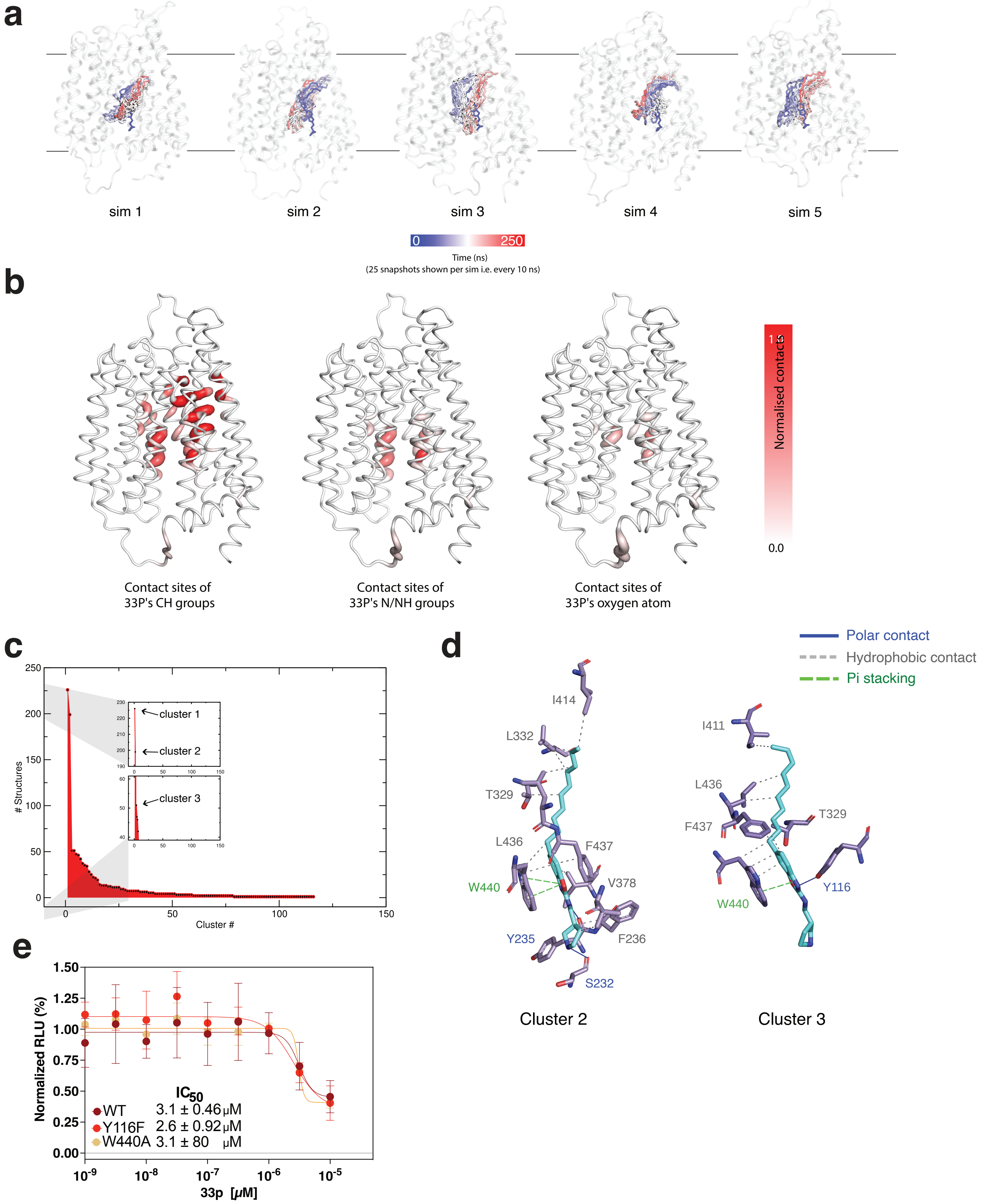

Supplementary Figure 8. Molecular dynamics of SPNS2 bound to 33p. a Time course of 33p location and pose for 5 simulations with 25 snapshots per simulations. **b** Frequency of contacts for residues within 4 Å of SPNS2. **c** Histogram of SPNS2 with 33p structural clusters. SPNS2 snapshots are clustered if within 1 Å RMSD. **d** 33p interactions with SPNS2 in clusters 2 and 3. Carbons of SPNS2 side chains and 33p are shown in purple and cyan, respectively. Interaction types are annotated by PLIP, with the interaction between 33p and Tyr116 identified in PyMol. **e** Inhibition of SPNS2 mutants Y116F and W440A by 33p (N = 8).

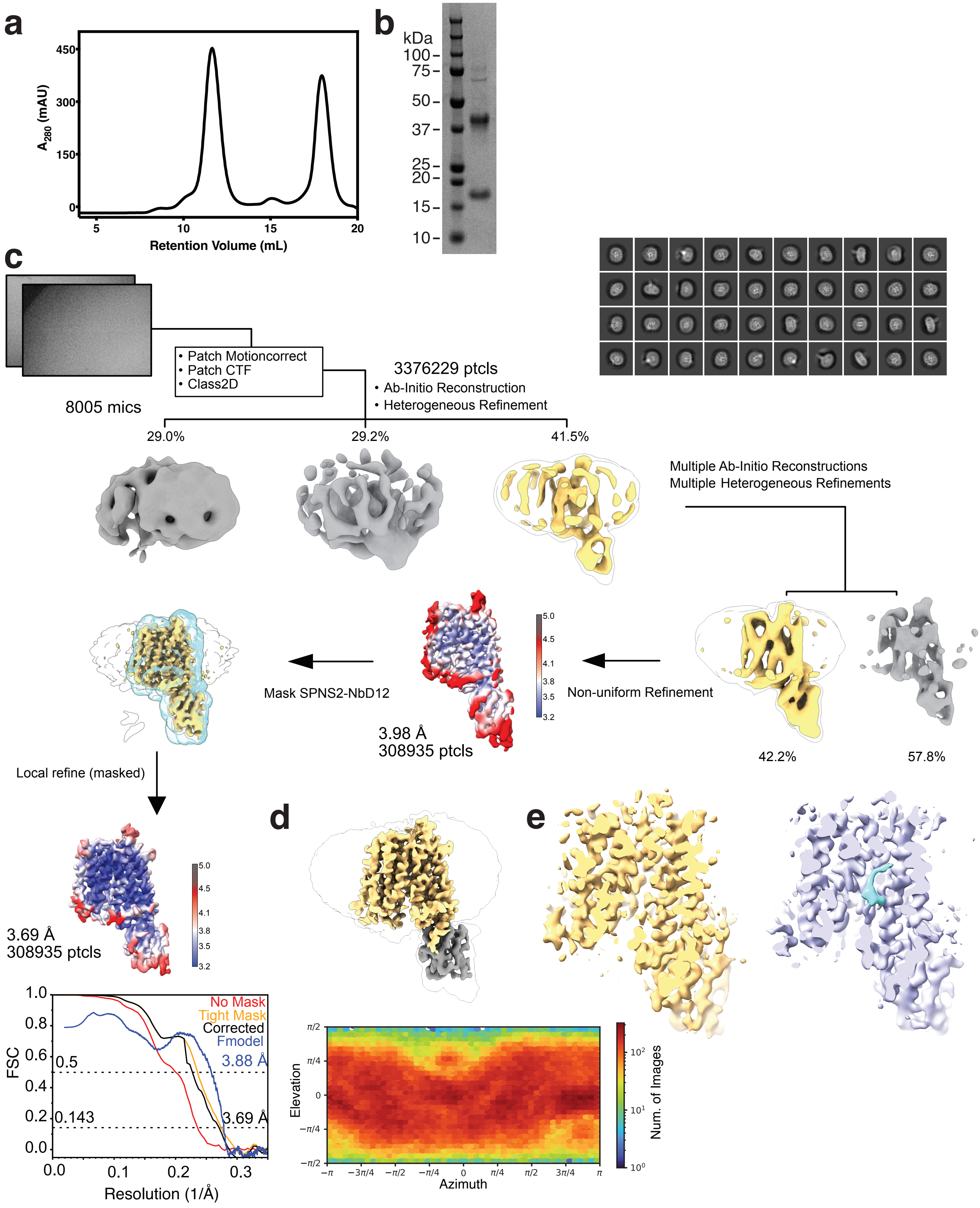

Supplementary Figure 9. Structure determination of SPNS2 in LMNG. **a** Preparative size-exclusion chromatography of SPNS2 in complex with NbD12 in LMNG on a Superdex 200 Increase 10/300 GL column. **b** Representative SDS-PAGE of SPNS2-NbD12 complex purification in LMNG from 10 biological replicates. **c** Flow chart for determining the structure of the SPNS2-NbD12 complex in LMNG. **d** Coulombic potential map for the SPNS2-NbD12 complex determined in LMNG. **e** Cross section through the binding site of SPNS2's Coulombic potential maps. CryoEM maps determined in LMNG and DDM are colored in yellow and purple, respectively, with the density for DDM is highlighted in blue.

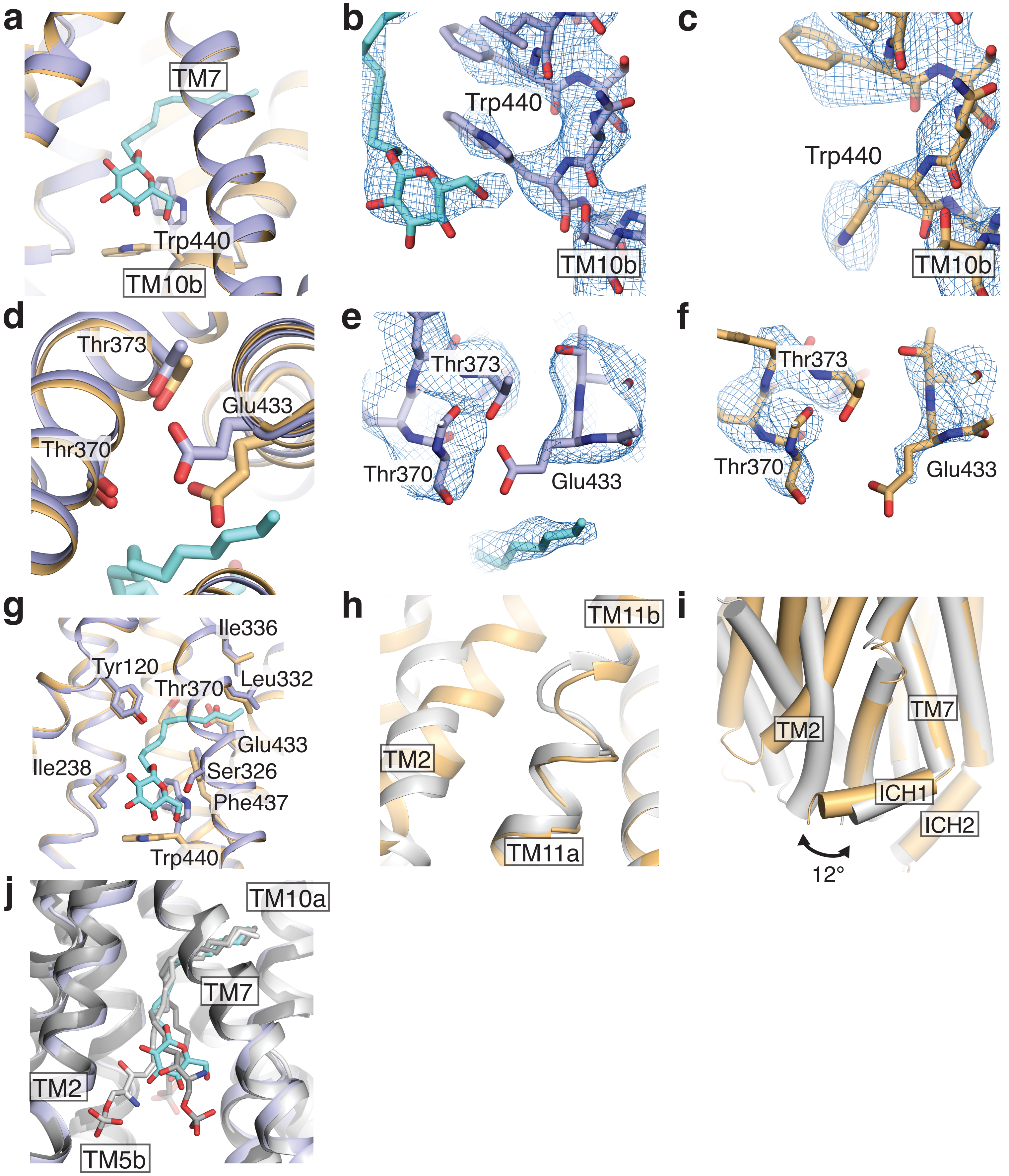

Supplementary Figure 10. Structural changes in SPNS2 upon substrate release or conformational change. a Structural alignment of SPNS2 in the presence and absence of bound DDM. Bound and apo structures are colored in purple and wheat, respectively. Coulombic potential map near Trp440 for (**b**) DDM bound and (**c**) apo SPNS2. **d** Movement of Glu433 in the DDM-bound and apo states. Coulombic potential map near Glu433 for (**e**) DDM bound and (**f**) apo SPNS2. **g** Overlay of the SPNS2 binding site in the DDM bound and apo states. Minimal structural changes are observed between the two states, except in the orientation of Trp440. **h** Change in secondary structure within TM11 between the inward-facing and outward-facing apo states. **i** Movement of ICH1 toward the transport domain in the inward-facing apo state. **j** Overlay of inward-facing SPNS2 structures with occupied binding sites (PDB: 7YUB, 8JHQ, 8EX4). DDM-bound and S1P-bound SPNS2 are shown in purple and grey, respecitvely. DDM and S1P are shown in cyan and grey, respectively.

Supplementary Table 1

CryoEM Data Collection, Processing, Refinement and Model Validation

	SPNS2-NbD12 in DDM	SPNS2-NbD12 in LMNG
EMDB id	EMD-18668	EMD-18667
PDB id	8QV6	8QV5
**Data Collection:**		
Microscope	eBIC Krios IV	eBIC Krios II
Camera	Gatan K3	Gatan K3
Voltage (kV)	300	300
Nominal Magnification	105,000	105,000
Pixel Size (Å/px)	0.829	0.831
Total dose (e/Å^2)	40.74	41.42
Defocus range (μm)	-1.0 to -2.4	-1.0 to -2.4
Movies collected	12,014	8680
**Image Processing:**		
Initial Particles	6,672,673	3,376,229
Final Particles	246,225	308,935
Map resolution (Å) GSFSC=0.143	3.68	3.69
**Refinement:**		
Resolution (Å)	3.7	3.7
Sharpening B-factor (Å^2)	-217.2	-211.1
Map CC (phenix CC mask)	0.83	0.80
Map-to-model resolution (FSC =0.5)	3.81	3.88
**Model validation:**		
Non-hydrogen atoms	4104	4080
Protein residues	551	548
Ligands	1	-
RMS bonds (Å)	0.002	0.002
RMS angles (°)	0.461	0.413
Molprobity Score	1.34	1.37
Clash Score	6.13	6.66
Rotamer outliers (%)	0.99	0.74
Ramachandran favoured (%)	98.34	98.70
Ramachandran allowed (%)	1.66	1.30

Supplementary Table 2

Details of simulation time, box dimensions, and numbers of lipid and solvent particles in each simulation condition.

		MD simulation systems		
		**SPNS2-S1P**	**SPNS2-FTY720P**	**SPNS2-33p**
**Simulation replicates x time**		5 x 250 ns	5 x 250 ns	5 x 250 ns
**Box dimensions (X, Y, Z nm^3) initially/after energy minimization**		10.3, 10.3, 12.19	11.26, 11.18, 12.3	10.3, 10.3, 12.19
**Box dimensions (X, Y, Z nm^3) after equilibration**		9.97, 9.97, 11.8	10.16, 10.09, 11.44	9.93, 9.93, 11.9
**Total number of atoms**		120,622	120,579	120,623
**Number of lipids**	Outer leaflet : POPC	134	134	134
	Inner leaflet : POPC	117	117	117
	Inner leaflet : $PI(4,5)P_2$	13	13	13
**Number of solvent molecules**	TIP3P water molecules	26,028	26,014	26,029
	Na^+ ions (incl. neutralizing ions)	108	109	108
	Cl^- ions	69	70	69
